# Riociguat, a soluble guanylate cyclase stimulator, ameliorates right ventricular contraction in pulmonary arterial hypertension

**DOI:** 10.1177/2045893217746111

**Published:** 2017-12-18

**Authors:** Mitsushige Murata, Takashi Kawakami, Masaharu Kataoka, Takashi Kohno, Yuji Itabashi, Keiichi Fukuda

**Affiliations:** 1 Department of Laboratory Medicine, School of Medicine, Keio University, Tokyo, Japan; 2 Department of Cardiology, School of Medicine, Keio University, Tokyo, Japan

**Keywords:** riociguat, right ventricular contraction, pulmonary hypertension

## Abstract

Riociguat is a soluble guanylate cyclase stimulator used for pulmonary hypertension (PH) treatment. We evaluated right ventricular (RV) contractile function in 27 PH patients receiving riociguat. A comparison of pre- and post-administration echocardiographic studies demonstrated significantly improved RV strain after riociguat treatment, even after adjusting for RV afterload.

## Introduction

Riociguat, a soluble guanylate cyclase (sGC) stimulator, was approved for treatment of chronic thromboembolic pulmonary hypertension (CTEPH) in 2013 and pulmonary arterial hypertension (PAH) in 2015. The previous studies demonstrated that treatment with riociguat led to a significant increase in 6-min walk distance (6MWD) as well as a decrease in pulmonary vascular resistance (PVR) and N-terminal prohormone of brain natriuretic peptide (BNP) in CTEPH^1^ and PAH.^2^ However, the mechanisms of such improvement of clinical condition remain to be clarified.

Marra et al. showed that treatment with riociguat reduced right heart size and improved tricuspid annular plane systolic excursion (TAPSE) and right ventricular (RV) S’ in patients with PAH and CTEPH.^3^ Furthermore, riociguat improved RV function in a murine model of secondary PAH induced by transverse aortic constriction.^4^ Interestingly, riociguat also improved left ventricular (LV) systolic function and LV interstitial fibrosis without any change in LV pressure overload, implicating the direct effect of riociguat on LV geometry and function. However, it remains arguable whether improvement of RV function by riociguat is attributable solely to the secondary effect of reduced RV pressure overload or to the additional effect of riociguat on heart contraction. In addition, quantifying RV function using conventional echocardiographic parameters remains difficult because of the complex geometry of the RV chamber.^5,6^ Recently, we demonstrated that a relatively new echocardiographic technology, speckle tracking echocardiography (STE), may be superior to conventional parameters for the evaluation of RV function.^7,8^ Thus, we aimed to investigate the effect of riociguat on RV contraction using STE in patients with CTEPH or PAH.

## Methods

The local ethics review committee approved the present study. We retrospectively evaluated 27 patients for the main analyses (seven with PAH, 20 with CTEPH; mean age = 66 years; 82% women). Echocardiography was performed before and after riociguat administration. Conventional RV parameters, such as RV diameter (RVD), TAPSE, RV S’, and RV fractional area change (RVFAC), were measured according to American Society of Echocardiography guidelines.^9^ RV longitudinal global strain (RVGLS) was measured using acoustic-tracking software (EchoPAC; GE Healthcare, Horten, Norway) as previously described.^10,11^ Pulmonary arterial systolic pressure (PASP) was estimated by adding the pressure gradient of tricuspid regurgitation and right atrial pressure estimated by inferior vena cava (IVC) diameter and respiratory change.

## Results

The mean baseline WHO classification and Borg score were 2.1 ± 0.8 and 4.0 ± 2.1, respectively. The mean riociguat dose was 7.3 ± 0.7 mg (range = 4.5–7.5 mg) and it was administered for 220 days on average. Eight patients were receiving other pulmonary vasodilator therapies. The mean BNP and 6MWD were 149 ± 323 pg/mL and 411 ± 104 m, respectively.

**Fig. 1.**
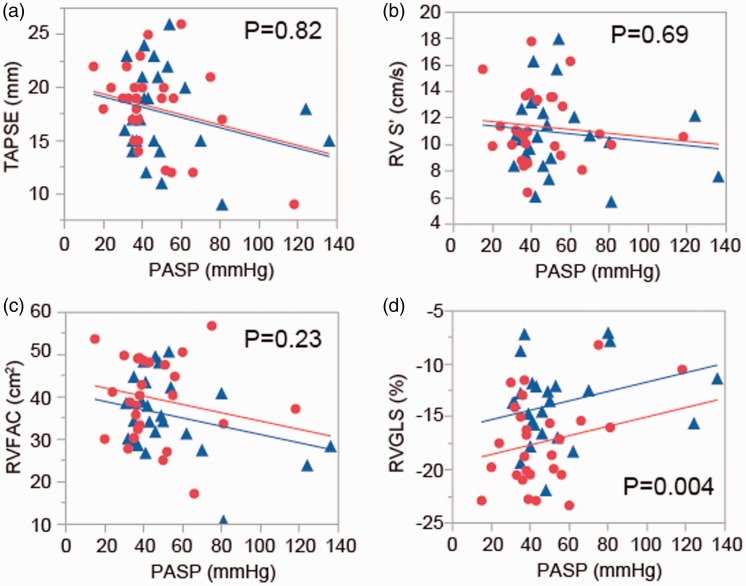
Analysis of covariance revealed no significant differences in TAPSE (a), RV S’ (b), or RVFAC (c) before (blue line) or after (red line) riociguat administration, after adjustment for PASP. In contrast, RVGLS was significantly improved after riociguat administration (d). PASP, pulmonary arterial systolic pressure; TAPSE, tricuspid annular plane systolic excursion; RVFAC, right ventricular fractional area change; RVGLS, right ventricular global longitudinal strain.

Table 1 shows a summary of the echocardiographic parameters before and after riociguat administration. PASP was significantly reduced after riociguat administration. RV remodeling was also ameliorated after riociguat administration as assessed by basal RVD and RV end-diastolic area index. Furthermore, riociguat administration significantly improved RV systolic function, including RVFAC and RVGLS, with no changes in TAPSE or RV S’. These results show the effects of riociguat on RV reverse remodeling as well as improvement of RV systolic function in patients with PAH, consistent with a previous report.^3^ Next, we performed analysis of covariance (ANCOVA) in order to investigate whether these improvements were solely due to the effect of reduced RV pressure overload. ANCOVA provided a comparison of RV function before and after riociguat administration under the same PASP. Interestingly, RVGLS was significantly better after riociguat administration, while the other RV parameters, including TAPSE, RV S’, and RVFAC, were not (Fig. 1). These results indicate that RV systolic function assessed by RVGLS was better after riociguat administration, even under the same PASP, implicating the improvement of RV contractile function by riociguat, regardless of RV loading.

**Table 1.** Echocardiographic parameters before and after riociguat administration.

Participants	Before riociguat	After riociguat	*P* value
IVC diameter (mm)	15.0 ± 3.2	13.8 ± 2.8	0.06
TRPG (mmHg)	50.3 ± 26.6	43.7 ± 20.5	0.004
ePASP (mmHg)	54.0 ± 26.6	45.7 ± 21.1	0.002
RAAI (cm^2^)	11.0 ± 3.2	10.1 ± 3.6	0.03
RVD1 (mm)	39.2 ± 5.6	36.1 ± 5.3	0.001
RVD2 (mm)	31.2 ± 5.2	28.6 ± 4.5	0.001
RVD3 (mm)	70.4 ± 8.1	65.9 ± 6.9	0.001
RVEDAI (cm^2^)	13.8 ± 3.0	11.9 ± 2.7	0.0005
RVESAI (cm^2^)	9.0 ± 2.7	7.2 ± 2.1	0.0002
RVFAC (%)	35.6 ± 9.0	39.6 ± 9.8	0.005
TAPSE (mm)	17.5 ± 4.4	18.1 ± 4.0	0.4
RV S’ (cm/s)	10.7 ± 3.1	11.4 ± 2.7	0.07
RVGLS (%)	–13.9 ± 3.7	–17.4 ± 4.1	0.0006
Tmax_SD (ms)	100 ± 36	87 ± 38	0.04

Data are given as the mean ± SD.

IVC, inferior vena cava; TRPG, tricuspid regurgitation pressure gradient; PASP, pulmonary arterial systolic pressure; RAAI, right atrial area index; RVD1, right ventricular basal diameter; RVD2, right ventricular mid diameter; RVD3, right ventricular longitudinal diameter; RVEDAI, right ventricular end-diastolic area index; RVESAI, right ventricular end-systolic area index; RVFAC, right ventricular fractional area change; TAPSE, tricuspid annular plane systolic excursion; RVGLS, right ventricular global longitudinal strain.

## Discussion

Ours is the first report demonstrating that riociguat can improve RV function as assessed by STE. RV contraction is affected by both RV afterload and RV contractility.^12^ Riociguat has a dual mode of action that sensitizes sGC to endogenously produced nitric oxide (NO) and increases sGC activity in the absence of NO, thereby improving hemodynamics in PAH patients with impaired NO-sGC-cyclic guanosine monophosphate signaling. Furthermore, recent studies demonstrated that riociguat ameliorated PAH due to left heart disease and prevented RV hypertrophy in experimental models of PAH.^4,13^ In addition, riociguat improved systolic function and reduced fibrotic tissue remodeling and degeneration.^13^ We found that even under the same PASP, RV strain was improved after riociguat administration compared to that measured before treatment, suggesting that riociguat may have an additional effect on RV contraction other than PASP reduction. Ishizu et al. reported that LV longitudinal strain could be a surrogate of subendocardial fibrotic change and may be useful for risk stratification of hypertensive heart failure.^14^ If this also applies to the right ventricle, riociguat may ameliorate fibrotic remodeling in patients with PAH, resulting in improved RV longitudinal strain. This finding is consistent with our data showing no treatment-related differences in TAPSE, RV S’, or RVFAC under the same PASP, as these parameters have no relationship with fibrotic change in the myocardium.

Our study population included patients with relatively mild PH (pre-riociguat mean PA pressure measured by right heart catheterization = 28.3 mmHg) and this cohort may show no significant improvement in TAPSE or RV S’ because these values were within normal limits at baseline. Importantly, however, even this population demonstrated impaired baseline RV strain; thus, RV strain could be a useful parameter to detect subtle changes in RV function.

In the clinical setting, we sometimes find that riociguat does not reduce mean pulmonary arterial pressure (mPAP) as much as expected, even in cases showing clinical improvement. This finding could explain why RV output increased due to the amelioration of RV systolic function in parallel with reduced PVR, resulting in a slight reduction of mPAP. In this regard, mPAP may not be a useful surrogate marker, and, as direct evaluations of RV systolic function are available, additional echocardiographic assessments might facilitate the management of patients with PAH. Among echocardiographic parameters, RV strain may be superior to conventional RV parameters, such as TAPSE and RV S’, for the evaluation of drug-induced effects in the same patients. However, the number of participants in this study was small and more extensive studies are currently ongoing.
